# Contrasting the seasonal and elevational prevalence of generalist avian haemosporidia in co‐occurring host species

**DOI:** 10.1002/ece3.6355

**Published:** 2020-05-27

**Authors:** Joshua G. Lynton‐Jenkins, Aisha C. Bründl, Maxime Cauchoix, Léa A. Lejeune, Louis Sallé, Alice C. Thiney, Andrew F. Russell, Alexis S. Chaine, Camille Bonneaud

**Affiliations:** ^1^ Centre for Ecology and Conservation University of Exeter Penryn UK; ^2^ Station d'Ecologie Théorique et Expérimentale (UMR5321) CNRS Université Paul Sabatier Moulis France; ^3^ Institute for Advanced Studies in Toulouse Toulouse France; ^4^ Present address: Max Planck Institute for Evolutionary Anthropology Leipzig Germany

**Keywords:** avian malaria, *Cyanistes caeruleus*, host generalist, *Leucocytozoon*, *Parus major*, *Plasmodium*, seasonality

## Abstract

Understanding the ecology and evolution of parasites is contingent on identifying the selection pressures they face across their infection landscape. Such a task is made challenging by the fact that these pressures will likely vary across time and space, as a result of seasonal and geographical differences in host susceptibility or transmission opportunities. Avian haemosporidian blood parasites are capable of infecting multiple co‐occurring hosts within their ranges, yet whether their distribution across time and space varies similarly in their different host species remains unclear. Here, we applied a new PCR method to detect avian haemosporidia (genera *Haemoproteus, Leucocytozoon,* and *Plasmodium*) and to determine parasite prevalence in two closely related and co‐occurring host species, blue tits (*Cyanistes caeruleus,* *N* = 529) and great tits (*Parus major*, *N* = 443). Our samples were collected between autumn and spring, along an elevational gradient in the French Pyrenees and over a three‐year period. Most parasites were found to infect both host species, and while these generalist parasites displayed similar elevational patterns of prevalence in the two host species, this was not always the case for seasonal prevalence patterns. For example, *Leucocytozoon* group A parasites showed inverse seasonal prevalence when comparing between the two host species, being highest in winter and spring in blue tits but higher in autumn in great tits. While *Plasmodium relictum* prevalence was overall lower in spring relative to winter or autumn in both species, spring prevalence was also lower in blue tits than in great tits. Together, these results reveal how generalist parasites can exhibit host‐specific epidemiology, which is likely to complicate predictions of host–parasite co‐evolution.

## Graphical abstract

Understanding the ecology and evolution of parasites is contingent on identifying the selection pressures they face across their infection landscape. Here, we detect avian haemosporidia (genera *Haemoproteus*, *Leucocytozoon*, and *Plasmodium*) and determine parasite prevalence in two closely related and co‐occurring host species, blue tits (*Cyanistes caeruleus*) and great tits (*Parus major*). We study changes in prevalence across an elevational gradient and between seasons. Our results reveal how generalist parasites can exhibit host‐specific epidemiology, which is likely to complicate predictions of host–parasite co‐evolution.

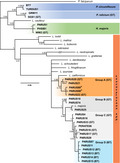

## 1 INTRODUCTION

Epidemiological dynamics and virulence evolution are dependent on the selective pressures that parasites face across their infection landscape (Alizon, Hurford, Mideo, & Van Baalen, 2009; Mackinnon & Marsh, 2010; Rigaud, Perrot‐Minnot, & Brown, 2010). These pressures will vary dependant on the host species infected (as hosts differ in their physiology, immunology, behavior, and distribution [Innes, 1997; Li, Cowles, Cowles, Gaugler, & Cox‐Foster, 2007; Palinauskas, Valkiunas, Križanauskiene, Bensch, & Bolshakov, 2009; Perkins & Swayne, 2001]), and on seasonal and spatial variation in abiotic conditions; some effects of which may be mediated through the host (Amundsen, Knudsen, Kuris, & Kristoffersen, 2003; Cosgrove, Wood, Day, & Sheldon, 2008; Tack, Thrall, Barrett, Burdon, & Laine, 2012). In parasite ecology, the existence of spatial and temporal structuring of parasite prevalence is well established (Altizer et al., 2006; Penczykowski, Laine, & Koskella, 2016). Climatic variability, such as over an elevation gradient or between seasons, is a particularly common driver of parasite prevalence because abiotic conditions (e.g., temperature) can influence parasite transmission and development (Harvell et al., 2002; LaPointe, Goff, & Atkinson, 2010; Sehgal, 2015). For example, sporogony (development of infective sporozoites) of the protozoan blood parasite *Plasmodium relictum* within its mosquito vector was shown to significantly decrease below 21°C (LaPointe et al., 2010). In addition, seasonal variation in infection risk coinciding with key phenological events, such as the onset of host reproduction and vector emergence, can give rise to temporal variation in prevalence (Altizer et al., 2006; Cosgrove et al., 2008). Rodent tick‐borne encephalitis infection dynamics, for example, are driven by the synchronous postwinter activity of larval and nymphal ticks, which is in turn determined by the rapidity of autumnal ground temperature declines (Randolph, Green, Peacey, & Rogers, 2000). Collectively, these abiotic and biotic variables sculpt intricate infection dynamics which are then likely made more complex for generalist parasites that infect multiple hosts. However, empirical studies of the spatial and temporal variation in prevalence of generalist parasites across multiple host species remain rare (Penczykowski et al., 2016; Rigaud et al., 2010).

Avian haemosporidia (encompassing the agents of avian malaria) are a species‐rich assemblage of blood parasites which often occur as complex communities within host populations (Harvey & Voelker, 2019; Oakgrove et al., 2014; Ricklefs et al., 2005; van Rooyen, Lalubin, Glaizot, & Christe, 2013b). The most prevalent genera are as follows: *Haemoproteus*, *Leucocytozoon*, and *Plasmodium* (Bensch, Hellgren, & Pérez‐Tris, 2009). Our ability to detect these parasites using molecular methods has enabled a better understanding of haemosporidian community ecology and epidemiology (Bensch et al., 2000, 2009; Hellgren, Waldenström, & Bensch, 2004; Waldenström, Bensch, Hasselquist, & Ostman, 2004), and recent studies have demonstrated the value of using avian haemosporidia to explore fundamental questions in parasitology (Drovetski et al., 2014; Hellgren, Pérez‐Tris, & Bensch, 2009; Jenkins, Delhaye, & Christe, 2015; Mata, da Silva, Lopes, & Drovetski, 2015). Haemosporidia adopt both host‐generalist and host‐specialist infection strategies, yet few studies have attempted to resolve how these host‐specific strategies might impact parasite ecology (Hellgren et al., 2009; Ventim et al., 2012). Climatic variation across an elevational gradient and throughout the year is predicted to impact the spatial and temporal distribution of haemosporidia, primarily via an influence on vector ecology and parasite development (Atkinson et al., 2014; Harrigan et al., 2014; LaPointe et al., 2010). For example, additional to the thermal requirements for *P. relictum* parasite development (i.e., shown by LaPointe et al., 2010), elevation has also been linked to host infection risk. In Hawaii, mosquito vectors decrease in numbers at high elevations such that populations of honeycreepers living above 1,000 m interact less with mosquitoes and have decreased exposure to *Plasmodium* (Atkinson & LaPointe, 2009). *Plasmodium relictum* prevalence in vectors has also been shown to vary seasonally, either in response to within‐vector competition between parasites or due to the availability of infected hosts (Lalubin, Delédevant, Glaizot, & Christe, 2013). However, many studies which identify these spatial and temporal patterns in haemosporidian prevalence have focused on either single host or parasite species, or have explored generalizations across the host or parasite community (i.e., by pooling infection data for host or parasite species) (Cosgrove et al., 2008; Huang, Dong, Zhang, & Zhang, 2015; Oakgrove et al., 2014; Pulgarín‐R, Gómez, Robinson, Ricklefs, & Cadena, 2018; Santiago‐Alarcon, Bloch, Rolshausen, Schaefer, & Segelbacher, 2011).

To examine the extent to which the spatiotemporal infection dynamics of generalist parasites is equivalent among host species, we surveyed haemosporidian parasites in populations of great tits (*Parus major*) and blue tits (*Cyanistes caeruleus*) during a three‐year period and across an elevational gradient in the French Pyrenees. These two host species are commonly infected by a diversity of haemosporidia, including representatives of the three major genera: *Plasmodium*, *Haemoproteus*, and *Leucocytozoon*, with community structure varying in both time and space (Glaizot et al., 2012; Jenkins & Owens, 2011; la Puente et al., 2010; van Rooyen, Lalubin, Glaizot, & Christe, 2013a; Schumm et al., 2019; Wood et al., 2007). Existing molecular detection methods, which amplify a region of the parasite's mitochondrial genome, are efficient at detecting *Plasmodium* and *Haemoproteus* (Hellgren et al., 2004). However, nontarget amplification has been reported to occur in blue tits where *Leucocytozoon* parasites also prevail (Cosgrove, Day, & Sheldon, 2006; Cosgrove et al., 2008). To address this issue, we designed a new amplification method aimed at specifically detecting *Plasmodium* and *Haemoproteus* infections to the exclusion of *Leucocytozoon* infections. In conjunction with a previous method designed for *Leucocytozoon* amplification (Hellgren et al., 2004), we surveyed the haemosporidian community with two main aims. First, we report the prevalence and diversity of haemosporidian parasites within our two host species, revealing genetically diverse parasites, which appear to adopt host‐specific or host‐generalist strategies. Next, while controlling for important infection predictors (i.e., host age), we identified whether the prevalence of generalist parasites varies similarly across season and elevation between the two host species.

## 2 MATERIALS AND METHODS

### 2.1 Study populations and sampling

We captured adult great tits (*N* = 443) and blue tits (*N* = 531) in the Ariège Pyrenees in France. Four study sites of nest box populations have been established within 14 km of one another close to the Station for Theoretical and Experimental Ecology in Moulis (42°57′29″N, 1°05′12″E) and cover an elevational range from 430 to 1,530 m. As expected, temperatures decrease with increasing elevation at our study sites (Bründl, 2018). These sites, their individual elevation ranges, contemporary nest box numbers, and positions are as follows: Moulis (430–593 m; 159 boxes; 42°57′90″N‐42°58′36″N, 01°05′31″E‐01°05′73″E), Cescau (549–1,091 m; 209 boxes; 42°55′34″N‐42°56′46″N, 01°02′47″E‐01°03′47″E), Galey (821–1,193 m; 105 boxes; 42°56′64″N‐42°57′24″N, 00°54′13″E‐00°55′30″E), and Castera (1,058–1,530 m; 147 boxes; 42°53′74″N‐42°55′07″N, 01°05′40″E‐01°03′43″E). With each 1,000 m increase in elevation, we could expect an approximate decrease in surface air temperature of 5.5°–6.5°C (Rolland, 2003). This variation is seen most starkly at temperature minima, for example, in 2016–2017 a difference of 5°C was recorded between the low elevation site Moulis (565 m, min to max = −7.6 to 32.1°C) and the higher elevations of the site Castera (1,502 m, min to max = −12.6 to 30.8°C). The landscape of this study is predominantly mixed deciduous woodland, interspersed by small patches of conifers and open fields used for low‐intensity pastoral farming.

Birds were caught and banded at these sites during the breeding (May and June) and nonbreeding (October–March) seasons (Bründl, 2018). During breeding, birds were captured in their nest boxes using spring traps, while mist nets were deployed near artificial feeders in the nonbreeding season. Individuals used in this study were captured between May 2015 and May 2017, and therefore, these data encompass three breeding seasons and two overwintering periods. On capture, morphological data were recorded (sex and age). Sex was determined based on plumage characteristics and the presence or absence of the brood patch. Because they are less sexually dimorphic, the sex of blue tits was not recorded for individuals captured outside of the breeding season. Individuals were categorized into either of two age classes: as yearlings or as postsecond year adults based on contrast between the greater and primary coverts (Svensson, 1992). On first capture, all individuals were fitted with a CRBPO metal identification ring and unique colored band combination. This allowed for the recording of recaptured individuals. A ~35 μl blood sample was obtained by brachial venipuncture. Blood samples were collected with sodium heparinized micro‐hematocrit capillaries (Hirschmann Laborgeräte) and then transferred to centrifuge tubes prefilled with ~1 ml of 96%–100% ethanol.

### 2.2 Molecular analysis

We extracted DNA from blood samples using the DNeasy Blood & Tissue extraction kit (QIAGEN^®^) following the manufacturer's protocol pertaining to nucleated blood. We standardized these extractions to working concentrations of 25 ng/μl. To detect haemosporidians, samples were subjected to two nested‐polymerase chain reaction (PCR) methods which target a specific region of the blood parasites’ mitochondrial cytochrome b (Cytb) gene. To amplify the DNA of *Leucocytozoon* spp., we followed the protocol developed by Hellgren et al. (2004), using primers and cycle conditions shown in Table S1.

To amplify *Plasmodium* and *Haemoproteus* DNA, we designed new primers based on published sequences of common *Plasmodium*, *Haemoproteus*, and *Leucocytozoon* species, some of which had previously detected in populations of our host species throughout Europe (Accession numbers: *P. relictum* (HM031937), *P. circumflexum* (JN164734), *P. cathemerium* (AY377128), *P. elongatum* (AF069611), *H. majoris* (JN164727), *H. multipigmentatus* (KY653756), and *Leucocytozoon* spp. (EU627797, FJ168563, and KX832559)(Bensch et al., 2000; Cosgrove et al., 2008; Glaizot et al., 2012; Jenkins & Owens, 2011). These primers were designed to be specific to *Plasmodium* and *Haemoproteus* sequences, while still encompassing the region of the apicomplexan's mitochondrial Cytb gene now widely used to classify these parasites (Bensch et al., 2000, 2009). As the primer binding regions selected here are conserved between *Plasmodium* and *Haemoproteus* species, these primers are suitable for amplifying a range of species within these genera (e.g., between three dissimilar species of haemosporidia: *P. cathemerium* (AY377128), *P. elongatum* (AF069611), and *H. multipigmentatus* (KY653756)) the primers only mismatch the target region by a maximum of 2bp). We adopted this approach as our previous application of *Plasmodium‐* and *Haemoproteus*‐specific primers (as found in Hellgren et al. (2004)) had led to unintentional amplification of *Leucocytozoon* DNA. This issue has been reported elsewhere (Cosgrove et al., 2006) and is likely most noticeable in our study population due to the very high prevalence rates of *Leucocytozoon* species (>90%). We concur with Cosgrove et al. (2006) that this unintended targeting of *Leucocytozoon* mitochondrial DNA is likely made possible by the lack of specificity in the first‐round forward primer (HaemNFI) and second‐round reverse primer (HaemR) which must then act in concert in the second round of PCR and enable amplification. Our solution has been to use two reverse primers considerably more specific to *Plasmodium* and *Haemoproteus* species such that no combination of forward and reverse primer could result in nontarget amplifications. Since applying this technique no unintended amplifications of *Leucocytozoon* DNA have occurred. Although others have recently tackled the issue of primer specificity in molecular detection (Ciloglu, Ellis, Bernotienė, Valkiūnas, & Bensch, 2019), our approach differs in facilitating the complete amplification of the parasite's mtDNA *cyt‐b* region as established by Bensch et al. (2000) for lineage determination.

Primers and cycle conditions for the *Plasmodium* and *Haemoproteus* targeted nested‐PCR are provided in Table S1. Both rounds of this nested‐PCR used reaction volumes of 25 μl. In the first round, around 25ng of total genomic DNA was combined with reagent proportions as follows: 1X DreamTaq Buffer (Thermo Fisher Scientific), 0.4 mM of each deoxynucleoside triphosphate, 0.56 μM of Plas1F and 0.04 μM of Plas1RP, and 0.625U DreamTaq DNA polymerase (Thermo Fisher Scientific). In the second round, 1 μl of first‐round product was combined with the following reagent proportions: 1X DreamTaq Buffer (Thermo Fisher Scientific), 0.4 mM of each deoxynucleoside triphosphate, 0.6 μM of both HaemFP and HaemRP, and 0.625U DreamTaq DNA polymerase (Thermo Fisher Scientific). Reactions were carried out in Applied Biosystems Veriti™ Thermal Cyclers and run with negative controls (sterile Milli‐Q water, 1/10 samples). Each reaction run included at minimum one positive control (DNA of verified positive infection status). Second‐round PCR products were separated on 2% agarose gels containing RedSafe™ Nucleic Acid Staining Solution (20,000×) (iNtRON Biotechnology Inc.) and ran at 100 V for 60 min before visualizing under UV. An approximately 500 bp (550 bp for Plasmodium/Haemoproteus PCRs) band indicated positive amplification and the presence of haemosporidians. Individuals with negative results were passed through the PCR process again to verify their status.

Nested‐PCR products which displayed successful amplification were purified in preparation for sequencing. Clean‐up reactions used Exonuclease 1 and Antarctic Phosphatase (New England BioLabs^®^) to degrade unutilized primers and hydrolyze excess dNTPs. Reactions were in volumes of 15 μl, containing: 12 μl of PCR round‐two product, 0.9 μl Milli‐Q water, 0.5 μl Antarctic Phosphatase (2.5 U), 1.5 μl Antarctic Phosphatase Buffer (10X), and 0.1 μl Exonuclease 1 (1 U). This mixture was incubated at 37°C for 40 min, heated to 80°C for 10 min, and then cooled to 4°C. Cleaned product was then diluted to nucleic acid concentrations of approximately ≤10 ng/μl, 5 μl of which was sequenced either bidirectionally (using both primers) or unidirectionally using the primers HaemFL (for *Leucocytozoon* amplicons) or HaemRP (for *Plasmodium/Haemoproteus* amplicons) (Eurofins sequencing service, Eurofins‐MWG).

Sequences were assembled, aligned, and analyzed using Geneious (Geneious^®^ 9.1.5, Biomatters, Ltd., New Zealand). Parasite lineages were identified by carrying out a BLAST search on the MalAvi database (Bensch et al., 2009). Sequencing results for *Leucocytozoon* lineages could at times not be completely resolved due to slightly shorter sequence reads or more commonly due to multiple infections preventing decisive identification. In most cases, however, it was possible to identify the clade or paraphyletic group of lineages to which the lineage or constituent lineages belonged. Multiple infections (infection by more than one lineage of haemosporidian) were identified, as they result in double‐peaks on the sequence chromatograms. We identified double‐peaks using the Heterozygote Plugin (Geneious^®^ 9.1.5), marking each contentious peak with the appropriate IUPAC ambiguity code. We then compared these sequences to a library of all previously detected haemosporidian lineage within our population, and through a process of elimination (exclusion of lineages due to the absence of their sequence signals on the target chromatogram), it was possible to determine the most parsimonious combination of lineages which would produce the peak pattern observed (Drovetski et al., 2014). Although it was often not possible to completely resolve these mixed‐infections to just two distinct lineages, in most cases we could still identify the presence or absence of the lineage groups common in our populations (e.g., *Leucocytozoon* lineages clustered into four groups).

### 2.3 Phylogenetic and statistical analyses

Two phylogenetic analyses were carried out to determine evolutionary relationships and to contextualize the diversity of parasite lineages detected within our populations. In the first phylogeny, all verified haemosporidian lineages from our sampling were included, and sequences trimmed and standardized to 390 bp. The first phylogeny used *Plasmodium falciparum* (a human malarial parasite; Accession no. AY282930) as the outgroup and included 13 additional *Leucocytozoon* morphospecies for which sequence data were available (*L. toddi*, AY762076; *L. mathisi*, DQ177241, *L. danilewskyi*, EU624137; *L. buteonis*, EF607293; *L. majoris*, AY393804; *L. caulleryi*, AB302215; *L. schoutedeni*, DQ676823; *L. fringillinarum*, JQ815435; *L. quynzae*, KF479480; *L. sabrazesi*, AB299369; *L. californicus*, KR422359; *L. neotropicalis*, MK103894; *L. grallariae*, MK103895). It was generated using a Bayesian reconstruction performed using the MrBayes plugin (Ronquist & Huelsenbeck, 2003) in Geneious and a GTR + I + G substitution model (recommended for our alignment by JModelTest [Darriba, Taboada, Doallo, & Posada, 2012]). Two runs were conducted, both of 10 million generations and with sampling set to every 200 generations. Following a “burn‐in” of 25%, the remaining trees were used to calculate posterior probabilities. This approach is used elsewhere in studies of avian haemosporidia (Jenkins & Owens, 2011; Oakgrove et al., 2014). Our second phylogeny was constructed to contextualize two focal *Leucocytozoon* clades detected in our populations within the genus as a whole. For this phylogeny, *P. relictum* (this study) was used as outgroup and all sequence data available for *Leucocytozoon* lineages were downloaded from MalAvi (version 2.4.2, downloaded December 9, 2019). After sequence cropping and removal of partial sequences 447 lineages remained, in addition to two new lineages detected in this study. Construction was performed using the same parameters as above.

All statistical analyses were conducted using R version 3.5.1 (R Core Team, 2018) in RStudio v0.99.902 (RStudio Team, 2017). We applied a two‐proportion *Z* test to identify significant differences in parasite prevalence between the two host species. We then tested for significant predictors of infection using six logistic regression models (logit function) with infection status by either *Haemoproteus* (*H. majoris* lineages), *Plasmodium* (*P. relictum* lineages), or *Leucocytozoon* group *(A and D lineages)* as response variables (Table 1 for *Leucocytozoon* groups). We opted not to analyze *Leucocytozoon* groups B and C, as they were both paraphyletic and some of their constituent lineages showed contrasting distributions within our host species. Of the 972 birds captures, 181 were captured more than once, although the vast majority were only recaptured once. Because few birds have repeat samples and when they do the number of repeats is modally 1, we elected to remove recaptures from the analyses and to perform more robust nonmixed modeling than attempting to control for a lack of statistical independence arising from repeat samples by fitting individual identity as a random intercept (Harrison et al., 2018). Host species, host age, capture season, capture elevation (continuous), capture year, and interaction terms between host species and elevation, host species and season, and season and year were included as explanatory terms. Terms were dropped if doing so reduced the Akaike information criteria (AIC) estimator by at least 2.0, improving model fit (Zuur, Ieno, Walker, Saveliev, & Smith, 2009).

**TABLE 1:** Prevalence of haemosporidian lineages detected in great tits and blue tits in our study population

	Lineage	Morphospecies	Great tits (443)		Blue tits (529)		*Z*‐test	
			Count	As %	Count	As %	*z*	*p*
**Haemoproteus**	PARUS1	*H. majoris*	20	4.5%	34	6.4%	−1.30	0.19
	WW2	*H. majoris*	10	2.3%	3	<1%	**2.28**	**0.02**
	PHSIB1	*H. majoris*	1	<1%	0	0%	1.09	1.73
	***H. majoris***	—	31	7%	37	7%	0.002	1.001
	***Total***	—	31	7%	37	7%	0.002	1.001
**Plasmodium**	TURDUS1	*P. circumflexum*	3	<1%	3	<1%	0.22	1.17
	BT7	*P. circumflexum*	0	0%	1	<1%	−0.92	0.36
	GRW11	*P. relictum*	7	1.6%	9	1.7%	−0.15	0.88
	SGS1	*P. relictum*	82	18.5%	43	8.1%	**4.82**	**<0.001**
	***P. relictum***	—	138	31.2%	79	14.9%	**6.05**	**<0.001**
	***Total***	—	141	31.6%	83	15.5%	**5.95**	**<0.001**
**Leucocytozoon**	PARUS20	—	26	5.9%	17	3.2%	**2.01**	**0.04**
	PARUS21	—	2	<1%	1	<1%	0.73	1.54
	PARUS22	—	289	65.2%	101	19.1%	**14.62**	**<0.001**
	PARUS89*	‐	1	<1%	1	<1%	0.13	1.1
	PARUS90*	—	3	<1%	2	<1%	0.65	1.48
	**Group A**	—	311	70.2%	121	22.9%	**14.79**	**<0.001**
	PARUS19	—	2	<1%	1	<1%	0.73	1.54
	PARUS25	—	5	<1%	3	<1%	0.97	1.67
	PARUS74	—	0	0%	1	<1%	−0.97	0.36
	**Group B**	—	65	14.7%	14	2.6%	**6.83**	**<0.001**
	PARUS4	—	2	<1%	5	<1%	−0.91	0.36
	PARUS16	—	117	26.4%	1	<1%	**12.47**	**<0.001**
	PARUS17	—	7	1.6%	0	0%	**2.9**	**0.04**
	PARUS18	—	9	2.0%	13	2.5%	−0.44	0.66
	PARUS33	—	9	2.0%	2	<1%	**2.42**	**0.02**
	PERATE06	—	1	<1%	3	<1%	−0.83	0.41
	**Group C**	—	300	67.7%	157	29.7%	**11.83**	**<0.001**
	PARUS11	—	2	<1%	9	1.7%	−1.83	0.07
	PARUS12	—	0	0%	17	3.2%	**−3.81**	**<0.001**
	PARUS13	—	1	<1%	79	14.9%	**−8.31**	**<0.001**
	PARUS14	—	1	<1%	115	21.7%	**−10.3**	**<0.001**
	PARUS15	—	3	<1%	14	2.6%	**−2.33**	**0.02**
	PARUS87	—	0	0%	1	<1%	−0.92	0.36
	**Group D**	—	15	3.4%	392	78.4%	**−22.26**	**<0.001**
	***Total***	—	418	94.4%	500	94.5%	−0.11	0.91

Note

Lineage names were identified using MalAvi (Bensch et al., 2009). “Count” refers to the total number of infections by that lineage or clade. “As %” provides the prevalence of that lineage or clade in each host species. Two novel lineages are indicated with an asterisk and italicized. For classifications in bold we provide totals including infections that were not identified to lineage, but which could be classified as belonging to that group, species, or genera. Significant *z* values in bold reflect host‐specific differences in parasites prevalence, shading indicates the more common host species; yellow for great tits, blue for blue tits.

## 3 RESULTS

### 3.1 Haemosporidian prevalence, diversity, and host specificity

**FIGURE 1:**
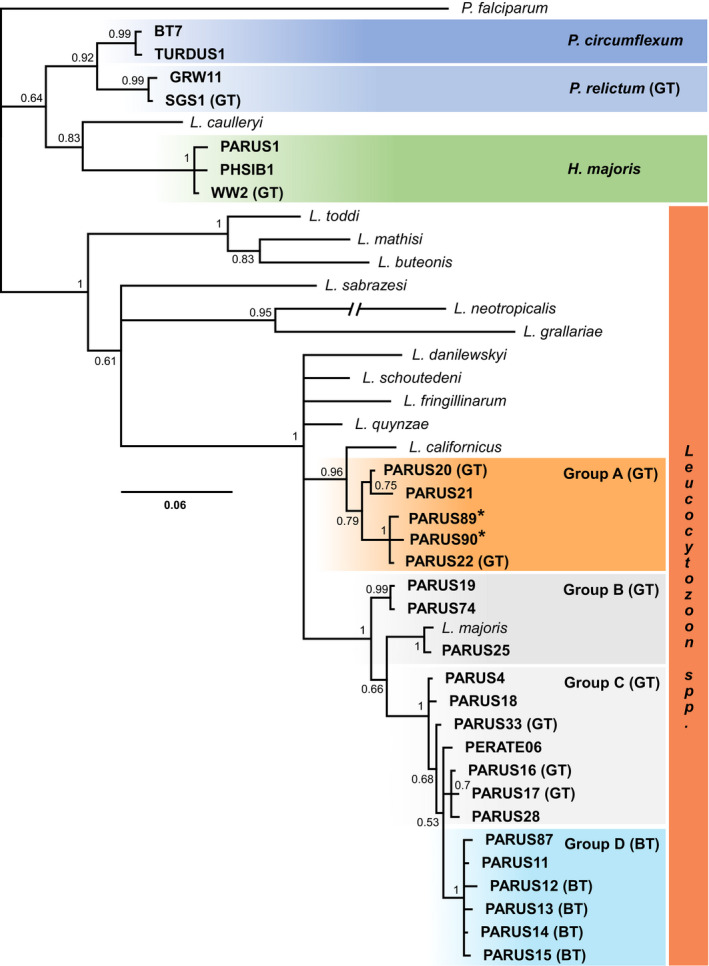
Consensus Bayesian phylogenetic tree of resident haemosporidian lineages (in bold) and 13 additional *Leucocytozoon* morphospecies lineages for context. Species names are provided in italics where known. Posterior probabilities shown on branches. Novel lineages are indicated with asterisks. Host preference is indicated for lineages, species, and groupings which had significant *z* values, (BT) for blue tits or (GT) for great tits. Color coding highlights the three genera: *Haemoproteus*, *Plasmodium*, and *Leucocytozoon* (Groups A–D). *Leucocytozoon* lineage groups received strong posterior probability scores and showed considerable within‐group sequence similarity. Double dash = 0.18 substitutions

**FIGURE 2:**
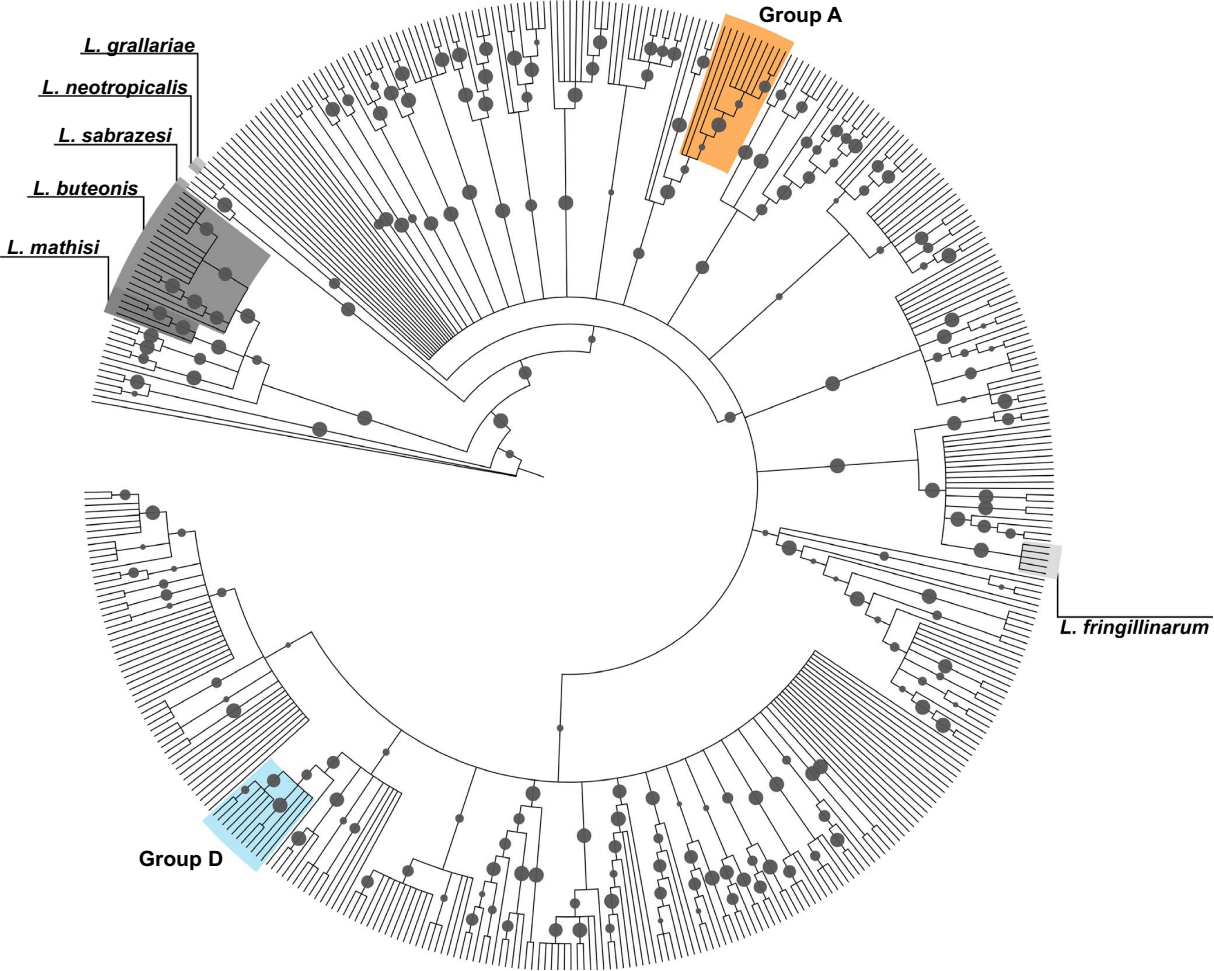
Consensus Bayesian phylogenetic tree of *Leucocytozoon* haemosporidian lineages available from MalAvi, rooted using *Plasmodium relictum*. Morphospecies included in the dataset are provided in italics. Resident *Leucocytozoon* lineages group A and D highlighted for comparison (we have highlighted the highest branching required to be inclusive of resident lineages, group A encompasses the undetected lineages: PARUS7, PARUS70, PERATE02, PARUS78, PARUS81, and CYACAE02, and group D includes the following: PARUS88, CCORAX02, GAGLA06, PARUS71, and PARUS84. Posterior probabilities ranging from 0.5 to 1 are represented on branches as scaled circles

In this study, we screened 972 samples from adult great (*N* = 443) and blue tits (*N* = 529). Haemosporidian infection prevalence was high in both species (98% for great tits and 96% for blue tits). *Leucocytozoon* parasites were the most prevalent, infecting >90% of individuals in both species. *Plasmodium* infections were mostly *P. relictum* (98%), which was the second most prevalent haemosporidian with higher rates of infection in great tits (32%) than in blue tits (16%). *Haemoproteus* prevalence reached 7% in both hosts, with all lineages attributed to the species *H. majoris*. Mixed infections were common. For example, 21% of birds were infected by both a *Leucocytozoon* and *Plasmodium* parasite, while 67% of *Leucocytozoon* infections were comprised of more than one lineage. In total, we detected 27 different haemosporidian lineages (23 in great tits and 26 in blue tits), including 20 *Leucocytozoon*, four *Plasmodium* and three *Haemoproteus* (Figure 1), and most lineages (78% of those detected) were found to infect both host species. We identified and verified two novel *Leucocytozoon* lineages. *Leucocytozoon* lineages were clustered into two clades and two paraphyletic groups (Figures 1 and 2, Table 1), with within‐group pairwise sequence identity ranging from 97% to 99.8% (i.e., 14–1 bp divergence). We termed these groups A–D, and while the utility of these classifications may not extend beyond our populations and remain to be described morphologically, they allowed us to classify the composition of mixed infections. Closely related lineages can represent within‐species variation rather than distinct species (Krizanauskiene et al., 2010; Palinauskas, Bernotienė, Žiegytė, Bensch, & Valkiūnas, 2017; Valkiūnas et al., 2019). *Leucocytozoon* groups A and D represent two clades of lineages with high within‐group pairwise sequence identity (group A = 97.43%, group D = 98.97%) and similar within clade lineage‐specific host prevalence's, as such we treated these two clades as distinct parasites in our analyses. We contextualized these two groups within the genus and revealed both that their divergence from one another was substantial (Figure 2). Prevalence for 12 of the 27 lineages and all four of the *Leucocytozoon* groups were significantly higher in one host, with most lineages (8 of 12) found at higher prevalence in great tits (Table 1). Only *Leucocytozoon* group D lineages showed a strong association with blue tits with a total clade prevalence of 78.4% versus 3.4% in great tits.

For all parasites, host age was found to be a significant predictor of infection status. For *H. majoris*, *P. relictum* and *Leucocytozoon* group A, older birds were more likely to be infected, while the opposite was true for *Leucocytozoon* group D. For example, *Leucocytozoon* group D prevalence decreased by 5% in postfirst year adults while group A prevalence was 14% higher. This increase in group A prevalence was more pronounced in blue tits than great tits, as older blue tits were more than twice as likely to be infected than first year birds.

### 3.2 Elevational and seasonal distribution of infections

**FIGURE 3:**
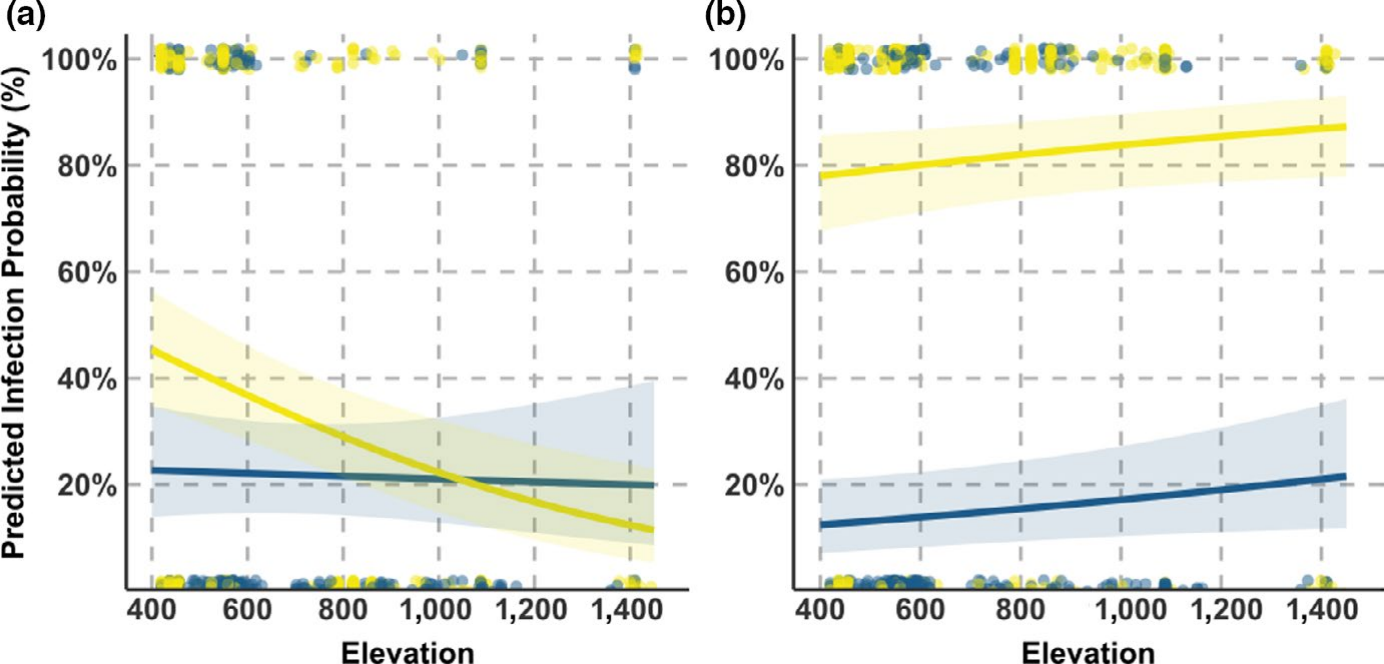
Predicted infection probabilities for two parasites in blue tits (blue) and great tits (yellow) across the elevational gradient; extrapolated from the models fitted in Table 2 for parasites where elevation was retained in the model. Plots are for (a) *Plasmodium relictum* and (b) *Leucocytozoon* group A

**FIGURE 4:**
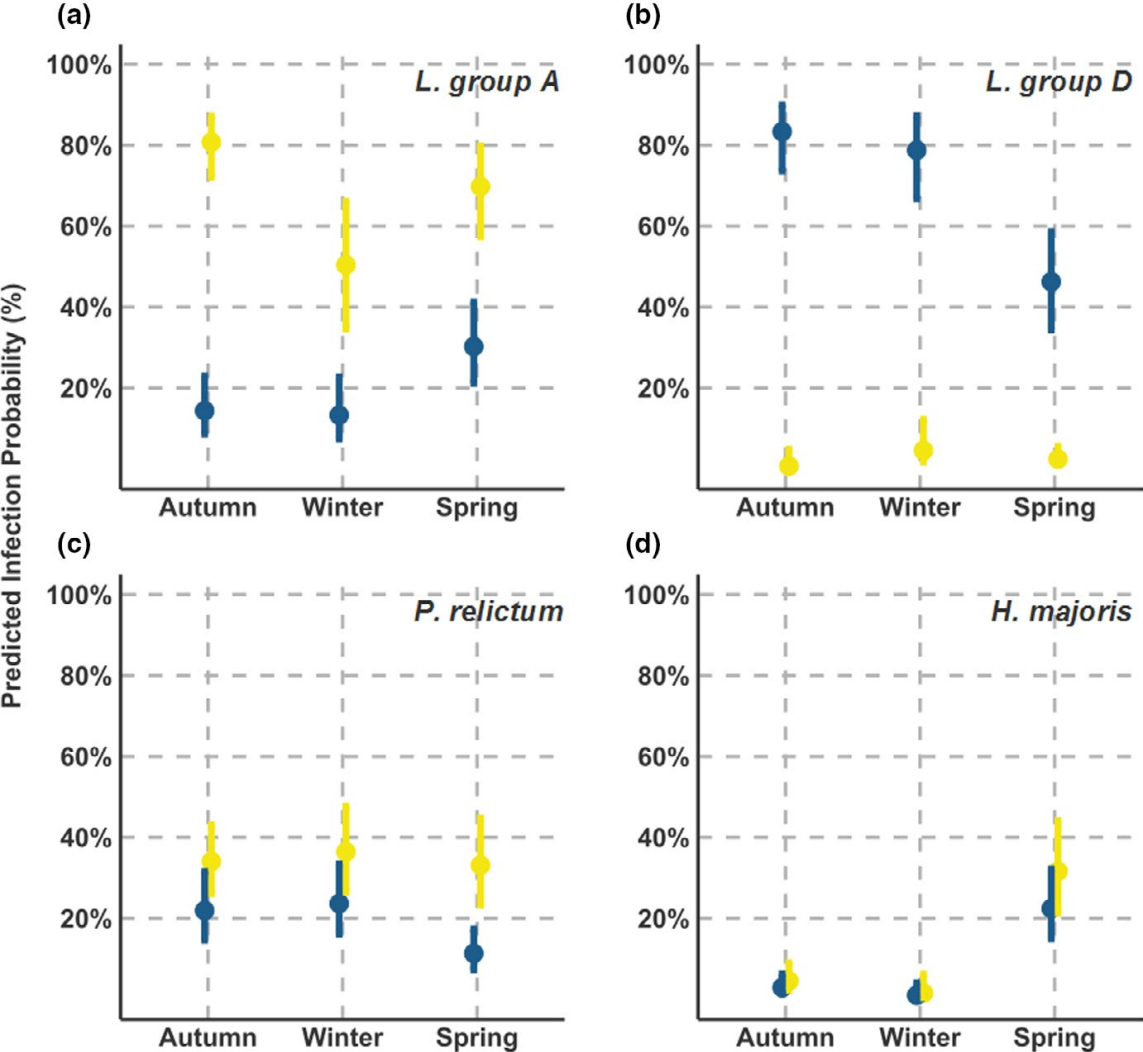
Predicted infection probabilities for four parasites in blue tits (blue) and great tits (yellow) dependent on season, extrapolated from models presented in Table 2. Plots show (a) *Leucocytozoon* group A, (b) *Leucocytozoon* group D, (c) *Plasmodium relictum*, and (d) *Haemoproteus majoris*

For two of the four parasites, capture elevation was retained in the models, although the direction of this effect was parasite specific (Table 2). Indeed for *Leucocytozoon* group A, infection probability increased at higher elevations (Table 2, Figure 3), while for *P. relictum*, fewer infections were found at higher elevations. Odds of infection decreased for great tits infected by *Plasmodium* by approximately 0.2% per meter of elevation, which equates to a 200% decrease across the 1,000 m elevational gradient at our study site. In three of the four parasites there was significant variation in infection prevalence between seasons (Table 2). For *H. majoris*, spring brought a substantial peak in infections, with prevalence recorded as: spring = 15%, autumn = 3%, and winter = 0.6%. Meanwhile, *P. relictum* prevalence was reduced in spring (14%) compared to autumn or winter (28%), although this was not determined to be significant (Figure 4c,d).

**TABLE 2:** Predictors of infection probability

	Term	Estimate	*SE*	OR	95% CI	*p*
*H. majoris*	***Age***	***0.65***	***0.28***	***1.91***	***1.11:3.29***	0.02*
	**Host (GT)**	**0.47**	**0.28**	**1.60**	**0.92:2.77**	**0.09**
	***Season: Spring***	***2.28***	***0.42***	***9.76***	***4.54:23.6***	<0.001*
	**Season: Winter**	**−1.11**	**0.80**	**0.33**	**0.05:1.36**	**0.17**
	***Year (2016)***	***−1.03***	***0.34***	***0.36***	***0.18:0.68***	0.002*
	***Year (2017)***	***−1.17***	***0.35***	***0.31***	***0.15:0.61***	<0.001*
	Elevation	0.0004	0.0005	—	—	0.50
	Host (GT) × Elevation	0.0002	0.001	—	—	0.86
	Host (GT) × Season: Spring	−0.46	0.89	—	—	0.60
	Host (GT) × Season: Winter	−0.66	1.65	—	—	0.69
*P. relictum*	***Age***	***0.54***	***0.17***	***1.71***	***0.06:0.36***	0.001*
	**Elevation**	**−0.0003**	**0.0006**	**0.9997**	**0.998:1.0008**	**0.60**
	***Host (GT)***	***1.75***	***0.48***	***5.77***	***2.26:14.72***	<0.001*
	**Season: Spring**	**−0.39**	**0.23**	**0.67**	**0.43:1.06**	**0.09.**
	**Season: Winter**	**0.14**	**0.22**	**1.15**	**0.74:1.79**	**0.53**
	**Year (2016)**	**−0.33**	**0.19**	**0.72**	**0.49:1.05**	**0.09.**
	***Year (2017)***	***−0.54***	***0.24***	***0.58***	***0.36:0.93***	0.02*
	**Host (GT)** × **Elevation**	**−0.001**	**0.0007**	**0.998**	**0.997:1.00007**	**0.06.**
	*Host (GT)* × *Season: Spring*	0.74	0.44	—	—	0.09
	*Host (GT)* × *Season: Winter*	0.006	0.42	—	—	0.99
	Season (Spring) × Year (2016)	0.41	0.49	—	—	0.40
	*Season (Winter)* × *Year (2016)*	−0.05	0.48	—	—	0.91
	*Season (Spring)* × *Year (2017)*	−0.48	0.53	—	—	0.36
	Season (Winter) × Year (2017)	na	na	—	—	na
*Leucocytozoon*	***Age***	***0.83***	***0.17***	***2.3***	***1.66:3.20***	<0.001*
*Group A*	**Elevation**	**0.0006**	**0.0003**	**1.0006**	***0.9999:1.0012***	**0.057.**
	***Host (GT)***	***3.22***	***0.36***	***25.03***	***12.59:52.85***	<0.001*
	***Season: Spring***	***0.95***	***0.38***	***2.59***	***1.23:5.59***	0.013*
	**Season: Winter**	**−0.09**	**0.48**	**0.91**	**0.38:2.21**	**0.84**
	**Year (2016)**	**−0.09**	**0.37**	**0.75**	**0.36:1.55**	**0.43**
	***Year (2017)***	***−0.29***	***0.37***	***3.55***	***0.18:7.41***	<0.001*
	***Host (GT)*** × ***Season: Spring***	***−1.55***	***0.45***	***0.21***	***0.09:0.51***	<0.001*
	***Host (GT)*** × ***Season: Winter***	***−1.33***	***0.45***	***0.26***	***0.11:0.63***	0.003*
	**Season (Spring)** × **Year (2016)**	**0.15**	**0.48**	**1.17**	**0.46:2.99**	**0.75**
	***Season (Winter)*** × ***Year (2016)***	***1.09***	***0.52***	***2.99***	***1.07:8.44***	0.04*
	***Season (Spring)*** × ***Year (2017)***	***−2.27***	***0.47***	***0.10***	***0.04:0.26***	<0.001*
	**Season (Winter)** × **Year (2017)**	**na**	**na**	**na**	**na**	**na**
	Host (GT) × Elevation	−0.0007	0.0006	—	—	0.29
*Group D*	***Age***	***−0.63***	***0.21***	***0.53***	***0.35:0.80***	0.003*
	***Host (GT)***	***−6.57***	***1.04***	***0.0014***	***0.00007:0.007***	<0.001*
	***Season (Spring)***	*−* ***1.76***	***0.39***	***0.17***	***0.08:0.36***	<0.001*
	**Season (Winter)**	**−0.30**	**0.43**	**0.74**	**0.31:1.74**	**0.49**
	**Year (2016)**	**−0.39**	**0.62**	**0.68**	**0.21:2.44**	**0.52**
	**Year (2017)**	**−0.45**	**0.41**	**0.63**	**0.28:1.41**	**0.27**
	***Host (GT)*** × ***Season: Spring***	***3.03***	***1.11***	***20.64***	***3.33:403.0***	0.006*
	**Host (GT)** × **Season: Winter**	**2.22**	**1.15**	**9.24**	**1.30:187.5**	**0.053**
	**Season (Spring)** × **Year (2016)**	**2.09**	**0.71**	**8.12**	**1.92:31.81**	0.003*
	**Season (Winter)** × **Year (2016)**	**−0.27**	**0.75**	**0.76**	**0.65:3.25**	**0.72**
	***Season (Spring)*** × ***Year (2017)***	***2.01***	***0.53***	***7.49***	***2.65:21.62***	<0.001*
	**Season (Winter)** × **Year (2017)**	**na**	**na**	**na**	**na**	**na**
	Elevation	0.0001	0.0005	—	—	0.68
	Host (GT) × Elevation	−0.0007	0.001	—	—	0.53

Note

Logistic regression results show the probability of infection by four focal parasites as a function of host species, host age category, season, elevation, and year. Host indicates host species, GT is great tit, blue tits served as the reference class. For season, autumn served as the reference class. For age class, first year birds (1) served as the reference class. And for year, 2015 served as the reference class. Terms retained in the final models are indicated in bold, and those significant at the <0.05 level are italicized and indicated with an asterisk. Odds ratio (OR) is provided for significant terms.

### 3.3 Host‐dependent distributions

For most parasites, we found significant host‐specific differences in infection distribution relating to either elevation or season. For *P. relictum*, as well as host‐dependent prevalence, we found that the decrease in prevalence at higher elevations was driven primarily by infections in great tits (Figure 3). Host‐specific differences in seasonal prevalence patterns were particularly striking; while the prevalence of *Leucocytozoon* group A in great tits decreased between autumn and spring, it increased in blue tits (Figure 4a). For group D, the opposite was true: prevalence in spring was greater in great tits relative to autumn (60% of group D infections in great tits were detected in the spring), while prevalence was unchanged in blue tits (Figure 4b).

## 4 DISCUSSION

Our analysis of avian haemosporidia infecting two co‐occurring and closely related host species reveals a shared community of generalist parasites, with 78% of lineages detected here present in both blue and great tits. Most of these generalist lineages have host‐specific prevalence, with significantly higher prevalence in one of the two hosts. While the elevational distributions of parasites were typically equivalent in both host species, seasonal prevalence patterns of multiple haemosporidia were host dependent. These findings suggest that distinct selective pressures may be encountered by generalist parasites across their host range.

Total haemosporidian prevalence was high in both host species, mirroring results reported for these birds across Europe; although there were also some parasite genus‐specific differences, which possibly reflected the unique ecologies of the study populations (Cosgrove et al., 2008; Glaizot et al., 2012; Jenkins et al., 2015; Jenkins & Owens, 2011; Knowles et al., 2011; van Rooyen et al., 2013a; Schumm et al., 2019; Tomás, Merino, Moreno, Morales, & Martínez‐De La Puente, 2007). *Leucocytozoon* lineages are elsewhere detected at high prevalence in similar montane tit populations (van Rooyen et al., 2013a, 2013b). Here, they were the most prevalent and diverse parasite taxa infecting both host species, adding to the body of molecular evidence demonstrating *Leucocytozoon* spp. (historically underrepresented in surveys of haemosporidia) to be important pathogens of *Paridae* throughout their European range (Jenkins & Owens, 2011; Knowles, Palinauskas, & Sheldon, 2010; van Rooyen et al., 2013a; Schumm et al., 2019). Morphological classification is still required for the majority of *Leucocytozoon* lineages being detected through molecular methods. This includes those detected here, which clustered into four clades/paraphyletic groupings, reflective of either deeper separation between groups of species or species with considerable within‐species variation. For example, group D lineages range from northern Europe to northwest Africa (Drovetski et al., 2014; Jenkins & Owens, 2011; Mata et al., 2015) and, as reported here, are found almost exclusively in blue tits (*Cyanistes* spp.). Mixed‐infections comprised of multiple lineages from this clade were common (40%) and between‐lineage sequence divergences were low (<1.1%, maximum of 4 bp difference between lineages) lending weight to the interpretation that group D may represent within‐species variation attributable to a highly host‐specific *Leucocytozoon* species. However, additional morphological and genetic analysis is required to confidently determine the biological relevance of these groupings (Nilsson et al., 2016; Sehgal et al., 2006; Valkiūnas, Sehgal, Iezhova, & Hull, 2010). Contrasting other studies of haemosporidia in these hosts, we found lower prevalence of both *P. relictum* and *H. majoris*, which may be driven by local vector abundance (Tomás et al., 2007). Studies conducted at lower altitude sites close to larger water bodies (ideal breeding grounds for mosquitoes; (Glaizot et al., 2012)) find *Plasmodium* prevalence to be at least twice that reported here for either blue tits (Knowles et al., 2011) or great tits (van Rooyen et al., 2013a). There is therefore considerable variation in haemosporidia prevalence for these host species across their ranges and additional to this, as shown here and by Schumm et al. (2019), variation also exists between the host species within a shared habitat.

We detected primarily generalist lineages (i.e., lineages detected in the two host species). There was not, however, equivalence in prevalence between host species, as 11 lineages and five pooled classifications (*P. relictum* and the four *Leucocytozoon* group) had significantly higher prevalence in one of the hosts. *Leucocytozoon* parasites showed a host preference that was lineage group (A–D) dependent. As previously mentioned, group D showed high host specificity for blue tits, with more than 20‐fold higher prevalence. Meanwhile *Leucocytozoon* lineages PARUS16 and PARUS17 in group C were almost exclusively found in great tits, and group A prevalence was three times higher in great tits than in blue tits. These patterns could represent differential exposure to vectors, however to our knowledge, no study has identified host preferences in relevant vectors (e.g., species of Culicidae, Simuliidae, or Ceratopogonidae) extending to such similar host species, nor between host species with such similar ecologies. As such, it seems more likely that host‐dependent prevalence of different lineage groups represents host specialization of the parasites per se. An interesting example is *Leucocytozoon* group A (predominately lineage PARUS22), which has a wide species range; eight host species across five genera (Bensch et al., 2009). This group is therefore a broad host generalist, yet we find here host‐bias in prevalence, with great tits more typically parasitized than blue tits. This finding is reminiscent of recent studies which have revealed haemosporidian generalists to have host‐species specific prevalence and infection intensities within their host ranges (Hellgren et al., 2009; Huang, Ellis, Jönsson, & Bensch, 2018).

Perhaps predictably, the haemosporidia we detected displayed distinct elevational and seasonal distributions as both are expected to play a significant role in the probability of infection. *Leucocytozoon* group A prevalence increased at higher elevations (in both host species) while *Leucocytozoon* group D prevalence did not change across the elevational gradient (Figure 3b). It seems reasonable to hypothesize that these two *Leucocytozoon* groups may be vectored by distinct *Simulium* spp. with opposing elevational distributions—indeed, such species have been recorded in the Pyrenees, but are not identified here (Clergue‐Gazeau, 1991). Similarly, the distinct genus‐specific seasonal prevalence patterns we detect may be driven by temporal variation in the emergence and activity peaks of the diverse fly species responsible for vectoring these parasites. However, this assumption requires validation, as vectors are rarely screened in studies of haemosporidia (Santiago‐Alarcon, Palinauskas, & Schaefer, 2012).

Host‐specific prevalence patterns across seasonal and elevational gradients are illustrative of the complex selective landscape parasites inhabit. We found more support for host‐dependent seasonal distributions than elevational ones. In terms of elevation, only *P. relictum* showed a host‐dependent distribution, where a decline in prevalence at higher elevations was more pronounced in great tits. This pattern possibly results from the sensitivity of this parasite to colder temperatures (LaPointe et al., 2010) and the minimal developmental temperature tolerances of its mosquito vectors (Atkinson et al., 2014; Pérez‐Rodríguez, Fernández‐González, De la Hera, & Pérez‐Tris, 2013). At low elevations, great tits are more parasitized, therefore where the environmental conditions for *P. relictum* transmission are more optimal (i.e., warmer climate), our hosts differ in their susceptibility. We cannot discern here whether this result stems from greater immunological susceptibility or higher transmission exposure (e.g., due to vector host bias) (Hellgren, Bensch, & Malmqvist, 2008; Palinauskas, Valkiunas, Bolshakov, & Bensch, 2008). In contrast to elevation, parasite seasonal prevalence patterns were often specific to the host species infected. For great tits, *Leucocytozoon* group A decreased in prevalence between autumn and spring, while in blue tits spring marked the peak in prevalence (Figure 4a). Similarly, the highly blue tit‐specific parasite *Leucocytozoon* group D was detected primarily in the spring for great tits; when prevalence decreased in blue tits (Figure 4b). Assuming these parasites are each transmitted by the same vector to both host species, these prevalence patterns are contrary to the expected (e.g., peak prevalence mirroring vector activity (Cosgrove et al., 2008)). They suggest instead that host‐specific infection dynamics may be driving population level prevalence rates. These dynamics could result from host‐specific environmental conditions; such as immune responses linked to seasonally variable conditions (Dowell, 2001), or from interactions occurring between coinfecting and host‐specific parasite communities (Lello, Boag, Fenton, Stevenson, & Hudson, 2004; Read & Taylor, 2001). Whatever the driver of these host‐specific seasonal prevalence patterns, they will likely be consequential for the trajectories of host–parasite co‐evolution in this system.

To conclude, by contrasting the complete haemosporidia community of two geographically overlapping bird species, we have revealed parasite host specificity apparent in both host preference and spatiotemporal prevalence patterns. Most haemosporidia were capable of infecting both bird species, and yet, despite the ecological and taxonomic proximity of the hosts, parasite prevalence was frequently host dependent. What causes these host preferences (i.e., host–parasite dynamics vs. vector‐driven) and the role they play in divergence between lineages (e.g., *Leucocytozoon* clades) could be fruitfully explored using a system such as ours. Understanding the mechanisms underlying these patterns would benefit from knowledge of vector host preferences, the timing of vector emergence, and the role of parasite–parasite interactions within hosts. Considering the high prevalence of co‐infection detected in this study, a future exploration that focuses on both multiple infections, as well as host‐specific dynamics, could provide promising insight into the ecology and epidemiology of complex host–parasite communities.

## CONFLICT OF INTEREST

None declared.

## AUTHOR CONTRIBUTIONS

**Joshua G. Lynton‐Jenkins:** Data curation (lead); formal analysis (lead); investigation (equal); methodology (lead); visualization (lead); writing – original draft (lead); writing – review & editing (equal). **Aisha C. Bründl:** Data curation (supporting); investigation (equal); project administration (supporting). **Maxime Cauchoix:** Data curation (supporting); investigation (supporting); project administration (supporting). **Léa A. Lejeune:** Data curation (supporting); investigation (supporting); project administration (supporting). **Louis Sallé:** Data curation (supporting); investigation (supporting); project administration (supporting). **Alice C. Thiney:** Data curation (supporting); investigation (supporting); project administration (supporting). **Andrew F. Russell:** Conceptualization (equal); data curation (supporting); funding acquisition (equal); methodology (supporting); project administration (equal); supervision (supporting); writing – original draft (supporting); writing – review & editing (supporting). **Alexis S. Chaine:** Conceptualization (equal); data curation (supporting); funding acquisition (equal); methodology (supporting); project administration (equal); resources (supporting); supervision (supporting); writing – original draft (supporting). **Camille Bonneaud:** Conceptualization (lead); formal analysis (supporting); funding acquisition (lead); investigation (supporting); methodology (supporting); project administration (lead); resources (equal); supervision (lead); writing – original draft (equal); writing – review & editing (supporting).

## ETHICAL APPROVAL

Capture of birds and blood collection was conducted under animal care permits from the French bird ringing office (CRBPO; n°13619; PP576) held by Dr. Alexis Chaine.

## Supporting information

Table S1

## Data Availability

The data that support the findings of this study are openly available in Dryad (https://doi.org/10.5061/dryad.b2rbnzsbf). Novel lineage sequences are available on GenBank (MN782320–MN782321) and have also been uploaded to MalAvi.
