# Supplementary material for: Contrasting the seasonal and elevational prevalence of generalist avian haemosporidia in co‐occurring host species
Source: Ecol Evol. 2020 May 27;10(12):6097–111. doi: 10.1002/ece3.6355 (PMC7319113; doi:10.1002/ece3.6355)
Supplement: Supplementary file 1 — Table S1 [file ECE3-10-6097-s001.docx]

**Table S1.** Nested-PCR protocol details for amplification of *Leucocytozoon*, & *Haemoproteus/Plasmodium* mtDNA targeting a region of the cytochrome b gene.

| **Method** | **1^st^ Round Primers** | | **Product length** | **Thermal Profile** | | | |
| --- | --- | --- | --- | --- | --- | --- | --- |
|  |  |  |  | **Step** | **Temperature** | **Duration** |  |
| Nested PCR: *Leucocytozoon* target (Hellgren et al. 2004) | Forward: | HaemNFI (5'-CATATATTAAGAGAAITATGGAG-3') | 617bp | Denature | 94 °C | 3 min |  |
|  |  |  |  | Denature | 94 °C | 30s |  |
|  |  |  |  | Anneal | 50 °C | 30s | **Ⱶ** 20 cycles |
|  | Reverse: | HaemNR3 (5'-ATAGAAAGATAAGAAATACCATTC-3') |  | Extend | 72 °C | 45s |  |
|  |  |  |  | Elongate | 72 °C | 10 min |  |
|  | | | | | | | |
| Nested PCR: *Haemoproteus/Plasmodium* target (this study) | Forward: | Plas1F (5'-GAGAATTATGGAGTGGATGGTG-3') | 706bp | Denature | 94 °C | 3 min |  |
|  |  |  |  | Denature | 94 °C | 30s |  |
|  |  |  |  | Anneal | 54 °C | 30s | **Ⱶ** 20 cycles |
|  | Reverse: | Plas1RP (5'-TACTCTCTTGCACCAAAAGC-3') |  | Extend | 72 °C | 45s |  |
|  |  |  |  | Elongate | 72 °C | 10 min |  |
|  | | | | | | | |
| **Method** | **2^nd^ Round Primers** | | **Product length** | **Thermal Profile** | | | |
|  |  |  |  | **Step** | **Temperature** | **Duration** |  |
| Nested PCR: *Leucocytozoon* target (Hellgren et al. 2004) | Forward: | HaemFL (5'-ATGGTGTTTTAGATACTTACATT-3') | 478bp | Denature | 94 °C | 3 min |  |
|  |  |  |  | Denature | 94 °C | 30s |  |
|  |  |  |  | Anneal | 55 °C | 30s | **Ⱶ** 35 cycles |
|  | Reverse: | HaemR2L (5'-CATTATCTGGATGAGATAATGGIGC-3’) |  | Extend | 72 °C | 45s |  |
|  |  |  |  | Elongate | 72 °C | 10 min |  |
|  | | | | | | | |
| Nested PCR: *Haemoproteus/Plasmodium* target (this study) | Forward: | HaemFP (5'- ATGGTGTTTTAGATATATGCATG-3') | 512bp | Denature | 94 °C | 3 min |  |
|  |  |  |  | Denature | 94 °C | 30s |  |
|  |  |  |  | Anneal | 58 °C | 30s | **Ⱶ** 30 cycles |
|  | Reverse: | HaemRP (5'-ATGTAAAGGAGTAGCATATCTATC-3') |  | Extend | 72 °C | 45s |  |
|  |  |  |  | Elongate | 72 °C | 10 min |  |
|  | | | | | | | |
